# Preoperative Neutrophil to Lymphocyte Ratio Predicts Complications After Esophageal Resection That can be Used as Inclusion Criteria for Enhanced Recovery After Surgery

**DOI:** 10.3389/fsurg.2022.897716

**Published:** 2022-07-13

**Authors:** Bo-Wen Shi, Li Xu, Chun-Xia Gong, Fu Yang, Yu-Dong Han, He-Zhong Chen, Chun-Guang Li

**Affiliations:** ^1^ Department of Thoracic Surgery, Changhai Hospital, Navy Military Medical University, Shanghai, China; ^2^ Department of Thoracic Surgery, Shanghai Pulmonary Hospital, Tongji University School of Medicine, Shanghai, China; ^3^ Department of Thoracic Surgery, Shanghai First People's Hospital, Shanghai Jiao Tong University School of Medicine, Shanghai, China; ^4^ Department of Thoracic Surgery, Affiliated Hospital of Qingdao University, Qingdao, China; ^5^ Department of Thoracic Surgery, Shanghai Chest Hospital, Shanghai Jiao Tong University, Shanghai, China

**Keywords:** preoperative, neutrophil to lymphocyte ratio, esophageal resection, complications, enhanced recovery after surgery

## Abstract

**Background:**

The neutrophil to lymphocyte ratio (NLR) has been reported as an indicator for poor prognosis in many cancers including esophageal cancer. However, the relationship between the NLR and postoperative complications after esophageal cancer resection remains unclear. At present, enhanced recovery after surgery (ERAS) lacks inclusion criteria. The aim of this study is to determine whether the preoperative NLR (_pre_NLR) can predict complications after esophageal cancer resection, which could represent the criteria for ERAS.

**Methods:**

This was a retrospective study on 171 patients who underwent esophagectomy at Hospital between November 2020 and November 2021(68 patients from Changhai Hospital, 65 patients from Shanghai General Hospital and 38 patients from Affiliated Hospital of Qingdao University). Univariate and multivariate logistic regression analyses were performed to demonstrate that the _pre_NLR could predict complications after esophagectomy.

**Results:**

A _pre_NLR cutoff value of 2.30 was identified as having the greatest ability to predict complications with a sensitivity of 76% and specificity of 65%. Moreover, the Chi-squared test results showed that the _pre_NLR was significantly associated with complications (x^2 ^= 13.641, *p* < 0.001), and multivariate logistic regression analysis showed that body mass index (BMI), p stage and _pre_NLR were independent variables associated with the development of postoperative complications (*p* < 0.05).

**Conclusion:**

The _pre_NLR can predict complications after esophagectomy, and these predicted complications can represent the criteria for recruiting patients for ERAS.

## Introduction

Esophageal cancer is the 6th leading cause of death from cancer and the 8th most common cancer in the world associated with high rates of morbidity and mortality; the 5-year survival is approximately 15%–25%, and the best prognoses are related to early diagnosis (1). Early detection and intervention can reduce adverse effects and improve the survival of patients. Although advancements in endoscopic techniques, such as endoscopic mucosal resection (EMR) or endoscopic submucosal dissection (ESD), have greatly benefited patients with early-stage disease, surgical resection remains the most important treatment measure. Esophagectomy is a complex procedure associated with complications such as pneumonia and anastomotic leakage that can restrict oral feeding, leading to short postoperative survival rates (2). With improvements in surgery and perioperative care resulting in reduced mortality after esophageal cancer (3), there is an urgent need to identify patients who are eligible for enhanced recovery after esophageal carcinoma resection. Enhanced recovery after surgery (ERAS) is based on a suite of clinical processes and evidence that it is on the premise of not increasing complications, it can improve comfort, shorten hospitalization time and reduce treatment costs. following a major surgery; it was initially established for colorectal surgery (4–6). Currently, ERAS has gained attention with regard to esophagectomy, and an increasing number of retrospective and prospective studies and clinical protocols are being reported. Accelerated recovery with ERAS is associated with not only reduction in the duration of hospital stay, pulmonary complications and anastomotic leakage but also decreased pain after surgery and faster return to normal life, especially oral feeding. However, to our disappointment, despite enhancement in the interventions during anesthesia and postoperative rehabilitation exercise, there is no consensus on when patients should return to oral feeding, as many patients suffer from anastomotic leakage during ERAS (7–11). Except for severe malnutrition and adverse reactions after neoadjuvant chemotherapy being considered in the exclusion criteria, there are no official guidelines to select the patients undergoing esophagectomy who are eligible for ERAS.

Systemic inflammatory responses have been demonstrated to be associated with cancer initiation and progression and poor outcomes in various malignant tumors (12–16). Inflammatory indicators such as the neutrophil to lymphocyte ratio (NLR) is suggested to include critical information that makes the NLR suitable as a serum biomarker that can reflect early complications associated with recovery, so that we may implement appropriate treatment strategies to address adverse outcomes in a wide range of patients (17–20). However, the relationship between preoperative or postoperative NLR and complications after esophagectomy is still uncertain. In this study, we aimed to determine the association between the NLR and postoperative complications to determine the potential of the NLR as an indicator for patients who are eligible for ERAS.

## Materials and Methods

### Patients and Perioperative Management

The work has been reported in line with the PROCESS criteria (21). This was a retrospective study, and a total of 668 patients who underwent video-assisted laparo-thoracoscopy *via* cervical anastomosis esophagectomy at three hospitals between November 2019 and November 2020 were recruited. The patients' eligibility for surgery was evaluated by a multidisciplinary team meeting following appropriate disease staging; meanwhile, physical fitness for surgery was evaluated by the operating surgeon and anesthetist. The exclusion criteria of this study were as follows: patients who underwent surgical procedures except video-assisted laparo-thoracoscopy *via* cervical anastomosis; patients who had advanced cardiopulmonary dysfunction; patients who used hemopoietin internally for two weeks; patients who had donated blood or underwent blood transfusion in the prior two weeks; patients who recently used glucocorticoids or human granulocyte colony stimulating factor; patients with unknown elevated or reduced white blood cell count and neutrophil or lymphocyte count; patients with hematological disease or autoimmune disease; patients with previous or concomitant cancer; patients who exhibited clinical evidence of infection or other inflammatory conditions; patients who used anti-inflammatory medicines within 1 week of surgery; patients with penicillin or cephalosporin allergy and who used other antibiotics during the operation; and patients suffering from bleeding, chylothorax, recurrent laryngeal nerve paralysis (RLNP) or pneumothorax. Ultimately, 171 patients were included in the study. All patients were subjected to white blood cell counting and neutrophil, lymphocyte, and albumin quantification within one week before surgery (the preoperative neutrophil to lymphocyte ratio was designated _pre_NLR). Samples were obtained on the day of surgery (_D0_NLR) and on the first day after surgery (_D1_NLR), and the data were included in the analysis (laboratory inspection was performed by the Experimental Diagnostic Department; reference ranges at our institution were 4.0–10.0 × 10^9^/L for white blood cell counts, 2.0–7.0 × 10^9^/L for neutrophil count, and 0.8–4.0 × 10^9^/L for lymphocyte count. The NLR was calculated by dividing the number of neutrophils by the number of lymphocytes).

The patients' clinical pathological parameters were collected from being admitted to hospital to being discharged. All patients had been given the same antibiotics at the induction of anesthesia to prevent infection and cure potential infections, enteral nutrition or total parenteral nutrition, proton pump inhibitor, and none of them were administered intravenous infusion of albumin during the study. The patients were routinely admitted to the intensive care unit after surgery. Warm water nasal feeding was performed the day after surgery; when patients felt uncomfortable, enteral nutrition was administered as early as possible to maintain normal physiology. X-ray of the chest and clinical characteristics (temperature, blood routine, drainage of chest tube) were used to evaluate eligibility to resume oral feeding. Informed consent was obtained from each patient, and the study procedures were approved by the Institutional Review Board.

### Pathological Analysis and Definitions

The data were obtained from hospital records and retrospectively analyzed. All recruited patients underwent pathological diagnosis *via* gastroscopy and biopsy before surgery. The American Society of Anesthesiologists (ASA) grade was assessed by the anesthetist in charge, and tumor size, invasion, operation time, intraoperative blood loss and other data were assessed by the surgeon in charge and the first assistant and recorded in the operation notes. Then, the specimens were sent for pathological examination after preservation in 10% formalin. Tumor staging was based on the American Joint Committee on Cancer (AJCC) Staging Manual (7th edition; Springer, New York) (22), combined with computed tomography scanning of the chest and abdomen. Complications were mainly defined according to mortality and morbidity, including pulmonary infection ((respiratory symptoms and infiltration on chest radiography associated with fever requiring antibiotic treatment), superficial or deep tissue infection, sepsis (23), anastomotic leakage and gastric fistula (based on video-assisted fluoroscopy or computerized tomography after oral contrast agent administration), anastomotic stricture, small bowel obstruction, postoperative, hemorrhage, arrhythmia, pneumothorax, and deep vein thrombosis). These complications were confirmed and recorded accurately by the doctor in charge.

### Statistical Analysis

We determined the optimal discriminator value for the NLR using receiver operating characteristic (ROC) curve analysis. An area under the curve (AUC) > 0.7 was considered clinically relevant. Chi-squared tests were used to compare proportions. Nonparametric variables were analyzed by the Mann–Whitney U-test. Multivariate logistic regression analyses were used to examine the effect of variables on the development of postoperative complications. p values were reported to three decimal places, and a two-tailed *p* value of <0.05 was taken as significant.

Statistical analysis was conducted with SPSS 22.0 (IBM Corporation, Armonk, NY, USA).

## Results

### Clinicopathological Features

A total of 171 patients (122 men and 49 women) met the inclusion criteria and were recruited for analysis; their age ranged from 45 to 76 years, with an average of 62.43 ± 6.60 years. The average body mass index (BMI) was 23.49 ± 2.86 kg/m^2^; the average operation time was 289.27 ± 56.22 min; the average blood loss was 225.50 ± 114.52 ml; and the average postoperative hospital stay duration was 11.11 ± 6.18 days. Twenty-one patients had complications, including respiratory infection in 5 (23.8%), anastomotic fistula in 7 (33.3%), catheter infection in 3 (1.8%), wound infection in 1 (4.8%), and other complications in 5 (36.3%) patients. None of the patients died during the operation. Meanwhile, 3 patients underwent re-intubation for pulmonary infection and respiratory failure.

### Relationship Between the NLR and Complications

**Figure 1:**
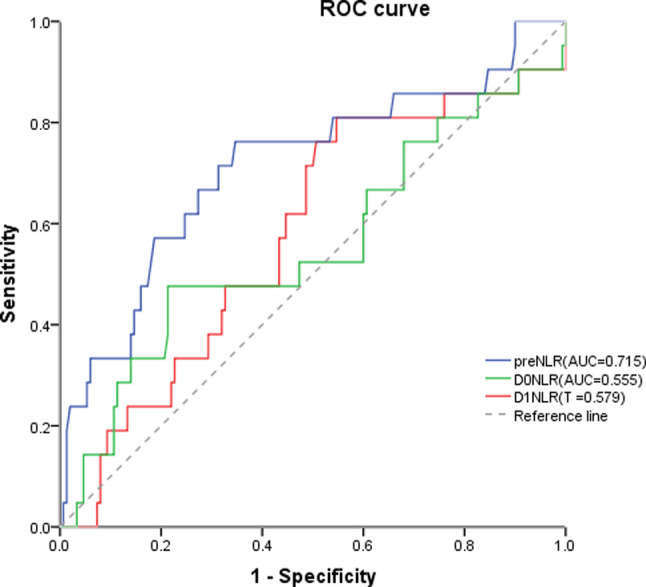
The preNLR showed the greatest discriminatory ability with an AUC of 0.72 (95% confidence interval 0.583–0.846, *p* = 0.001). A preNLR cutoff value of 2.30 was identified as having the greatest ability to predict complications with a sensitivity of 76% and specificity of 65% preNLR, pre-operative neutrophil to lymhocyte ratio; DONLR, the day of surgery neutrophil to lymhocyte ratio; DINLR, the first day after surgery neutrophil to lymphocyte ratio.

The _pre_NLR showed the greatest discriminatory ability with an AUC of 0.72 (95% confidence interval 0.583–0.846, *p* = 0.001). A _pre_NLR cutoff value of 2.30 was identified as having the greatest ability to predict complications (Figure 1) with a sensitivity of 76% and specificity of 65%. The average _pre_NLR was 2.45 ± 1.36.

### Relationship Between Clinicopathological and Complications

The Chi-squared test and Mann–Whitney U-test results showed that BMI (x^2 ^= 8.794, *p* = 0.003), ASA grade (x^2 ^= 7.735, *p* = 0.021), operation time (x^2 ^= 4.485, *p* = 0.034), intraoperative blood loss (x^2 ^= 3.909, *p* = 0.048, tumor size (x^2 ^= 3.909, *p* = 0.048), T stage (x^2 ^= 10.552, *p* = 0.014), N stage (x^2 ^= 12.028, *p* = 0.007), p stage (x^2 ^= 28.117, *p* < 0.001), postoperative hospital stay (z = −7.257, *p* < 0.001) and _pre_NLR (x^2 ^= 13.641, *p* < 0.001) were significantly associated with complications. There were no significant difference regarding age, sex, neoadjuvant chemotherapy, forced expiratory volume 1 (FEV1), albumin, or histological differentiation (*p* > 0.05) (Table 1).

**Table 1:** Relationship between preoperative neutrophil to lymphocyte ratio and clinicopathological characteristics in patients undergoing esophagectomy for esophageal cancer (*n* = 171).

	Uncomplicated	Complicated	Test Value	*p*-value
Gender				
Male	111	18	fisher	0.292
Female	39	3		
Age				
≤65	103	13	0.386	0.534
>65	47	8		
BMI				
≤24	106	8	8.794	0.003
>24	44	13		
ASA				
I	15	1	7.735	0.021
II	114	12		
III	21	8		
Neoadjuvant chemotherapy				
0	143	21	fisher	0.599
1	7	0		
FEV_1_			2.166	0.151
FEV_1_/_predict_FEV1			−1.509	0.131
Albumin			−0.165	0.869
Operation time				
≤240	106	10	4.485	0.034
>240	44	11		
Intraoperation blood loss				
≤250	110	11	3.909	0.048
>250	40	10		
Tumor size				
≤3	104	10	3.909	0.048
>3	46	11		
Tumor location				
Up	23	0	3.866	0.145
Middle	92	16		
Low	35	5		
T				
1	67	4	10.552	0.014
2	36	4		
3	43	12		
4	1	1		
N				
0	104	9	12.028	0.007
1	33	5		
2	10	6		
3	3	1		
P-Stage				
I + II	122	6	28.117	0
III + IV	27	15		
Histological differentiation				
High	20	1	1.259	0.533
Moderately	111	17		
Poorly	19	3		
Hospital stay			−7.257	0
NLR				
≤2.3	110	7	13.641	0
>2.3	40	14		

### Multivariate Logistic Regression Analysis of Clinicopathologic Characteristics in Patients with Complications

The results of univariate analysis for factors associated with postoperative complications after esophagectomy are shown in Table 2. We next analyzed the clinicopathologic characteristics for which Chi-squared test and Mann–Whitney U-test results showed *p* < 0.15. In addition to BMI, ASA grade, operation time, intraoperative blood loss, tumor size, T, N, and p stage, postoperative hospital stay and _pre_NLR, we included FEV1/predicted FEV1 (z = −1.509, *p* = 0.131) and tumor location (z = 3.866, *p* = 0.145) in our analyses. Indeed, BMI, p stage and _pre_NLR were independent variables associated with the development of postoperative complications (*p* < 0.05).

**Table 2:** Clinicopathologic characteristics in patients undergoing Esophagectomy: MultivariateLogistic Regression Analysis.

Clinicopathologic characteristics	B	S.E.	Wald	OR	95% CI	*p*-value
BMI	1.515	0.582	6.789	4.551	1.456–14.228	*p* < 0.05
p-Stage	2.491	0.585	18.104	12.075	3.83–38.043	*p* < 0.05
preNLR	1.527	0.572	7.131	4.605	1.501–14.126	*p* < 0.05

## Discussion

To the best of our knowledge, this is the first study demonstrating that the NLR (in addition to surgical factors) is a potential indicator of complications in patients after esophageal cancer resection.

In the past years, several studies have demonstrated that the Glasgow Prognostic Score (GPS) (the combination of an elevated C-reactive protein (CRP) level and hypoalbuminemia) is associated with poor survival in various cancers (24), but as the primary limitation, CRP level is not a normal examined factor in many institutions. Until now, the systemic inflammatory prognostic marker, the NLR, has received great interest because it is convenient, inexpensive and easy to evaluate by clinical doctors (25). Neutrophils infiltrate in tumor tissues that produce high levels of vascular endothelial growth factor (VEGF). Neutrophils can also produce carcinogenic element to stimulate tumor cells to secrete VEGF. High levels of VEGF can provide a suitable environment for tumor growth and proliferation and promote the formation of tumor blood vessels, thus accelerating tumor growth (26); additionally, a decrease in lymphocytes in the tumor stroma can contribute to tumor cell growth and migration and is associated with poor prognosis (27). The NLR is proven to be an effective predictive factor of prognosis in many malignant tumors (28–30). An increasing number of studies have demonstrated that inflammation plays a critical roles in malignant tumor progression and prognosis; in other words, the regulation of inflammation may benefit patients by preventing the formation of tumors and improving outcomes (31, 32). A study demonstrated that pneumonia has a negative impact on overall survival after esophagectomy, and strategies to prevent pneumonia after esophagectomy can improve outcomes after this operation (33). Several studies demonstrate that inflammation is associated with the prognosis of esophageal cancer. A study from Japan on gastric carcinoma demonstrated that the _pre_NLR can independently predict the development of postoperative infection-associated complications and reduced survival after gastrectomy. An elevated NLR could trigger postoperative infection and increase the risk of recurrence in patients with these postoperative complications after gastrectomy (34). An study from UK on sixty-five patients showed that an elevated NLR as early as 48 h following surgery was a highly sensitive and significant predictor of postoperative complications (35). One meta-analysis from China comprising nine studies containing 2,513 patients concluded that a high NLR might represent poor prognosis and unfavorable clinicopathologic characteristics for patients with esophageal squamous cell carcinoma (ESCC) (36). These results suggest that the _pre_NLR might be a precise indicator of the balance between the tumor inflammatory response and host immune response.

In this study, we first analyzed the relationship between the NLR and main complications after esophagectomy. We investigated factors that had never been studied in this context before. To eliminate the confounding effect of the operation technique, we excluded patients with complications such as postoperative bleeding, recurrent laryngeal nerve injury and pneumothorax. To eliminate the effect of injury from different surgical procedures, we excluded a large number of patients who underwent Ivor-Lewis esophagectomy, nonendoscopic esophageal cancer resection and some other surgical procedures. Additionally, we excluded patients who had taken glucocorticoids, erythropoietin, or human granulocyte colony stimulating factor, patients who recently underwent blood donation or transfusion or patients with hematological diseases or autoimmune diseases to prevent the effect or routine changes in blood. Our study demonstrated the NLR along with BMI and p stage is an independent risk factor for complications. BMI has been proven to be a risk factor of complications and prognosis after resection in many cancers (37, 38). As far as we know, BMI is related to postoperative complications of tumor in literature reports. Patients with low BMI are prone to suffer complications such as poor incision healing, due to poor nutrition status. In addition, when serious complications occur, there may rise more mortality due to poor tolerance. Patients with high BMI are at higher risk of complications such as lung infection and deep vein thrombosis due to poor activity or high blood lipid levels (39, 40). NLR is an indicator of immune status and infection in tumor microenvironment. In many literature reports, NLR can predict infection, anastomotic fistula and other complications. Meanwhile, in our study, it was also found that patients with high NLR are at high risk of postoperative complications, mainly anastomotic fistula, pulmonary infection and catheter infection. Therefore, we believe that BMI can reflect the nutritional status and wound healing ability of tumor patients after surgery, but cannot completely predict the postoperative complications of esophageal cancer. Therefore, compared with BMI, NLR can predict the occurrence of non-operative complications after esophageal cancer more exactlly.

In this study, we analyzed the _pre_NLR, _D0_NLR and _D1_NLR and found that the _pre_NLR was most significant indicator for the occurrence of complications. We consider that the NLR after operation is likely highly influenced by the inflammatory reaction, and thus, it is unable to accurately indicate the condition of the patient. Our univariate analysis revealed that complications after esophagectomy are related to BMI, ASA grade, operation time, intraoperative blood loss and tumor stage. For patients undergoing thoracic surgery, postoperative rehabilitation exercise is important for promoting lung function recovery and can reduce complications such as pneumonia. Patients with high BMI have difficulty reaching effective cough and sputum production and performing early activities, and thus, these patients are likely to develop pneumonia and deep vein thrombosis. Because the operation time is much longer for esophagectomy than for other surgeries and because single lung ventilation is used, anesthesia can aggravate injury to the body, especially the lung, and is more likely to cause pneumonia and other complications.

Overall, this study has some limitations. We need to perform multicenter observational studies to prove that the _pre_NLR is an objective indicator of complications after esophagectomy, and it may be much more appropriate to introduce reference indicators such as CRP level and interleukin levels for comparison. Then, we can proceed with survival analysis and prognostic assessment. Additionally, we can find whether non-infection complications are associated with poor prognosis.

Importantly, in most cases, the indicators are present long before the complications can be identified. Therefore, we can use these indicators as early predictive markers. As esophagectomy is a complicated and traumatic procedure, more careful evaluation and intervention are needed to improve the postoperative outcomes. ERAS is a patient-centered, surgeon-led protocol combining anesthesia, nursing, nutrition and psychology. During recent years, ERAS has obtained much attention in the field of cancer resection (41, 42). ERAS can benefit patients by reducing the discomfort of tube detention, promoting quicker recovery to the physiological status, and reducing hospital stay duration and cost. ERAS is not commonly implemented in esophagectomy because of the complexity of the surgical procedure and high morbidity associated with postoperative complications, which have limited the progress in esophagectomy.

In the treatment of traditional mode, patients need to wait for about 1 week after radical surgery for esophageal carcinoma that they can get through eating liquid, removing the chest tube and gastric tube, that is lack of individual treatment differences. However, the patients expected to have good recovery do not need 1 week of fasting, why can’t we help that patients recover from surgery more quickly. According to the concept of ERAS, early removing the gastric tube and chest tube, early eating through the mouth can improve the comfort of patients, shorten the length of hospital stay, reduce the cost of treatment, faster recovered from surgery. But perfotming the implement ERAS such as early remove the chest tube and the gastic tube will not benefit for that patients with anastomotic fistula and infection to recovery, it can even pose a fatal risk, increasing complications and mortality. According to our opinion, we hope that NLR can be used to seclect that patients with esophageal cancer who are suitable for ERAS, so as to facilitate the rapid recovery safely. In the experimental design, we have excluded surgical complications such as bleeding and chylous leakage, patients with high NLR and unsuitable for ERAS will be delayed for feeding and removing the chest tube, in case of avoid serious chest infection or even death due to blind eating and chest tube removal.

The primary factors that influence the time to oral feeding and tube removal are pneumonia, anastomotic leakage and pleural effusion. If we can detect the indications for complications, we can identify patients undergoing esophagectomy who are suitable for ERAS, and this can significantly enhance recovery and reduce the cost after surgery; additionally we can implement early interventions to address postoperative complications.

## Conclusion

The _pre_NLR as an early predictor of complications can reduce postoperative mortality and improve survival rates. Herein, we consider preNLR can be used as inclusion criteria for enhanced recovery after surgery.

## Data Availability

The original contributions presented in the study are included in the article/Suplementary Material, further inquiries can be directed to the corresponding author/s.
